# Economical Auto Moment Limiter for Preventing Mobile Cargo Crane Overload

**DOI:** 10.3390/s20216355

**Published:** 2020-11-07

**Authors:** Soo-Hoon Noh, Yong-Seok Lee, Sang-Ho Kim, Jae-Sang Cho, Chang-Soo Han, Seung-Yeol Lee, Dong-Eun Lee

**Affiliations:** 1 Daihung Heavy Industries, Hongseon-gun 32280, Korea; nosh416@naver.com; 2 Daegu Gyeongbuk Institute of Science & Technology, Division of Intelligent Robotics, Daegu 42988, Korea; yslee2020@dgist.ac.kr (Y.-S.L.); shkim83@dgist.ac.kr (S.-H.K.); syl@dgist.ac.kr (S.-Y.L.); 3 Rich & Time, Seoul 08389, Korea; jscho@rntime.com; 4 Department of Interdisciplinary Robot Engineering Systems, Hanyang University, Ansan 15588, Korea; 5 School of Architecture, Civil, Environment and Energy Engineering, Kyungpook National University, Daegue 41566, Korea

**Keywords:** auto moment limiter, rollover prevention, cost effectiveness, mathematical model of cargo crane

## Abstract

This study presents a computational method called economical auto moment limiter (eAML) that prevents a mobile cargo crane from being overloaded. The eAML detects and controls, in real time, crane overload without using boom stroke sensors and load cells, which are expensive items inevitable to existing AML systems, hence, being competitive in price. It replaces these stroke sensors and load cells that are used for the crane overload measurement with a set of mathematical formula and control logics that calculates the lifting load being handled under crane operation and the maximum lifting load. By calculating iterative them using only a pressure sensor attached under the derrick cylinder and the boom angle sensor, the mathematical model identifies the maximum descendible angle of the boom. The control logic presents the control method for preventing the crane overload by using the descendible angle obtained by the mathematical model. Both the mathematical model and the control logic are validated by rigorous simulation experiments using MATLAB on two case instances each of which eAML is used and not used, while changing the pressures on the derrick cylinder and the boom angle. The effectiveness and validity of the method are confirmed by comparing the outputs obtained by the controlled experiments performed by using a 7.6 ton crane on top of SCS887 and a straight-type maritime heavy-duty crane along with eAML. The effects attributed to the load and the wind speed are quantified to verify the reliability of eAML under the changes in external variables.

## 1. Introduction

**Figure 1:**
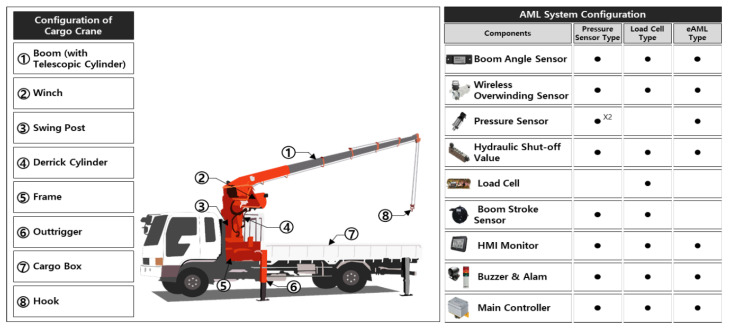
Cargo crane and auto moment limiter (AML) system configurations.

A mobile cargo crane is a construction equipment that locates construction objects using hydraulic pressure. It is equipped with crane machines in the vehicle’s loading box, thereby manifesting high mobility at a construction jobsite. The mobile cargo crane consists of a boom, a frame, a swing post, a winch, a derrick cylinder, a telescopic cylinder, and outriggers (Figure 1). These components are elaborated in another publication (UNIC 2020). The mobile cargo crane performs operations (e.g., adjusting outriggers, rotating the swing reducer, ups and downs of the boom by manipulating the derrick cylinder, intruding and extruding the boom by manipulating the telescopic cylinder, rise and fall of the hook by manipulating winch, etc.) by using hydraulic pressure.

It has superior productivity in lifting and moving heavy objects. However, accidents frequently occur because the crane operation and the manipulation of the crane machine components rely on the personal experience and judgment of the equipment operator. For the 11 year period of 1984 through 1994, the US Occupational Safety and Health Administration (OSHA) investigated 502 deaths in 479 incidents involving cranes in the construction industry [1]. The government agency has enforced regulations to install the auto moment limiter (AML) mandatory as a preventive measure against the crane rollover accident. Several protective safeguards have been developed as well [2,3,4,5,6] including methods that identify rollover danger zones using a mathematical relationship of the forces acting on the cargo crane components and dynamics in a rollover by considering the location and the travel path of a load [7]. This method improves the crane rollover safety by using a torque limiter [8,9] that automatically controls or stops the boom extrusion and crane rotation [10] using the threshold value of the rollover moment calculated by using multi-dimensions (i.e., boom length and angle) measured by multi-sensors. Consequently, any overload is excluded using wide-angle pin-type load cells, which complement the disadvantage of existing pin-type load cells capable of measuring only a narrow range [11] and calibrates the load moment limiter with a test bench [12] that communicates with the AML device using multi-sensors [13]. The abovementioned mainly complement the performance of existing sensors and rollover prevention systems [14,15,16,17,18,19,20,21].

Note that the prices of existing AML systems are relatively higher compared to those of mobile cargo cranes. In particular, the mobile cargo cranes mounted on vehicles are mainly owned and operated by small self-employed owners, and the expensive cost of existing AML systems is not favorable to them. A few manufacturers have attempted to make the overload prevention device available at an affordable price by changing the AML device components [22]. To address the issue, they save manufacturing costs legally by manipulating the pressure switch. The pressure switch measures the pressure of the fluid inside the derrick cylinder. It is not possible to measure the pressure because the fluid in the lower part of the derrick cylinder moves to the tank when the boom downs. Indeed, it is difficult to measure the overload on the boom attributed to the momentary and transitory changes in the dynamic moment caused by the change in the boom angle of the mobile cargo crane. Certainly, the conventional pressure sensor type AML system may measure the pressure on the bottom of the derrick cylinder only when the crane is static stop state intermittently. Noteworthy is that stopping motion to check for overload means the productivity delay; measuring the pressure in the dynamic lifting state means significant risk taking. Mobile cranes operated with the existing overload preventive devices expose significant risks to jobsite safety. It would be beneficial for the construction community to develop a new AML method that increases price accessibility and secures crane safety in responding to momentary changes in dynamic moments associated with crane operation, because the existing AML methods, which depend on multi-sensors, require a high cost. The cost reduction in the new AML system obtained by reducing the number of sensors measuring the length, angle, and load of a boom, which are cost items making an existing AML expensive, contributes to the encouragement of using the new device. Indeed, it may contribute to securing crane operation safety and improving construction productivity.

The research was conducted in five steps. First, the state-of-the-arts in the existing AML methods were investigated to identify new distinctive research contributions through a literature review. Second, the major issues limiting the performance of existing methods were identified. Third, the computational method called economical auto moment limiter (eAML), which computes the lifting loads, was implemented into an automated method using MATLAB. Fourth, the algorithms that estimates the maximum lifting load and controls the eAML were elaborated upon. Fifth, a detailed illustration of eAML was demonstrated using a case study. The validity of the method were verified by performing rigorously a series of simulation experiments that confirms if eAML prevents overload given virous load magnitudes and wind speeds. Finally, the research contributions and limitations are discussed on top of the experiment outputs involved in the overload measurements. The material in this paper is organized in the same order.

## 2. State-of-the-Art in AML Based on Maximum Lifting Load Prediction

Existing crane research proposes methods that evaluates the complex crane operation in the perspective of time, cost, productivity, and safety by using either 4D modeling, rule-based reasoning, mixed-integer programming, and/or agent based simulation independently or jointly. They include the calculation of utilization rates for planning crane deployment at job site [23], the identification of potential spatial conflicts by evaluating the collision possibilities associated with the multiple tasks, cranes, material supplies, and overlapping areas [24], the investigation of the dynamic effect of supply selection on crane efficiency [25], the optimization of crane location on its total costs of lifting operations to minimize operation costs [26], the optimization of the crane allocation in near real time by calculating crane movements checking for collisions [27], the improvement of the efficiency and safety of constraint-free crane path planning by handling the nature of dynamic constraints [28], the selection of cranes types and locations by detecting clash scenarios [29], and the quantification of the effect attributed to potential conflicts among the working cranes on the overall time and cost of crane operations [30]. Indeed, they consider the time, cost, and productivity in the crane operation primarily but handle accident events (i.e., clash or collusion, etc.) as a subsidiary concern. A few safety studies handle diverse aspects of accident factors. After introducing the sociotechnical model using 25 critical factors out of comprehensive 56 factors [31], the list was extended to 59 factors, hence, accommodating comprehensive accident phenomenon. Sadeghi et al. [32] identified the comprehensive risk factors that may be categorized into regulatory bodies, stakeholders, management of construction site during work process, workers and staff on job site, environment and equipment, and risks associated with mobility by conducting extensive literature review. Indeed, the failure modes and risk profiles involved in crane equipment and attachment include as follows: the quality and reliability of crane safety device (i.e., brake, limiter, protection device, etc.), assembling auxiliary tools (i.e., truck-crane, wire rope, installation tools, etc.), attachment device (i.e., welds, bolts, embedded parts, adhering bars, etc.), foundation components (i.e., supporting structure, concrete base, tension piles, etc.), crane structural components, and ergonomics of operator cab. It is well accepted that the limiter, called AML, is only a factor out of 59 risk factors in the crane operation risk directory [32].

To implement a corresponding measure to cope with each accident factor, diverse preventive measures, models, and methods have been proposed using several high techs. They include 3D motion capturing method avoiding spatial conflicts [33], high-definition cameras mounted on unmanned aerial vehicles to complement blind lifting attributed to reduced visibility of the crane operator, and 3D collision-free safe zone identification [34]. However, few studies propose a mathematical method to cope with the failure mode of moment limiter. The only failure mode of eAML is the fall of the boom that may cause the loss of control and the damage to the winch. The AML is a safety controller (i.e., limiter) that prevents a crane from overloading. It controls the rated allowable load that momentarily varies with the changes in boom length and angle. The existing AMLs may be classified into two classes (i.e., pressure sensing and tension sensing methods) according to the method by which the lifting loads are detected (Figure 1, [35]). The pressure sensing method prevents overload during load lifting by detecting the pressure on the lower part of the derrick cylinder. Meanwhile, the tension sensing method, which uses load cells, prevents overload by measuring the tension in the wire connected to the load. Existing AMLs comprise a combination of a pressure sensor attached to an up-and-down cylinder, an angle sensor attached to the boom, an extrusion length sensor attached to the boom, pressure sensors detecting the boom motions, an AML status monitor, a harness, an AML controller, and a load cell. In short, they use several sensors, which leads to unfavorable prices for small self-employed owners and less accessibility.

The new AML method reduces several sensors (e.g., distance sensors detecting the extruded length, pressure sensors for detecting the boom motion, load cells, etc.) that are essential for existing methods. It uses an algorithm that implements a new mathematical model predicting the overload occurring in mobile cargo cranes. The left-hand side of Figure 1 illustrates the cargo crane configuration. Existing AML methods require pressure sensors on the top of the derrick cylinder and length sensors on the boom. In contrast, the new AML does not, except for a pressure sensor under the bottom of the derrick cylinder. Existing load cell-type AML methods are expensive because they need load cells and length sensors. Given the price of sensors, the new AML method saves 81% and 83% in price compared to AMLs utilizing pressure sensors and road cells, respectively. Table 1 presents the advantages and disadvantages of the existing AMLs and the eAML. Both the pressure and tension sensing methods are weak in durability, high in price, and subject to load shell deformation. The eAML replaces the performance of the existing expensive AMLs by implementing control algorithms that utilize a mathematical model along with the input of the tube pressure sensors attached to the bottom of the derrick cylinder and the boom. The eAML achieves low-cost benefits by eliminating the usage of high-cost load cells and rod pressure sensors attached to the upper part of the derrick cylinder and by using short-length durable sensors. Note that the scope of this study is not to treat all of these factors using sensors, but to develop eAML that performs boom operations equivalently to those of existing AMLs by replacing expensive sensors (i.e., stroke sensors and loadcells) with low-cost pressure sensor, angle sensor, mathematical formula, and control algorithms. The eAML prevents the fall of the boom, the loss of control, and the damage to the winch by identifying the overload, while the boom is being manipulated to lift a weight. It is well accepted that the limiter, called AML, is only a factor out of 59 risk factors in the crane operation risk directory [32]. The only failure mode of eAML is the fall of the boom that may cause the loss of control and the damage to the winch. Certainly, the only risk is assuring the reliability of crane safety device (i.e., limiter) that manipulates the boom of mobile crane safely, because the purpose of this study is replacing the expensive stroke sensors and load cells with the economical sensors, formula, and algorithm.

## 3. Computational Method of the Allowable Lifting Load Using the Pressure on the Derrick Cylinder and the Boom Angle

### 3.1. Formulation of the Mathematical Model of the Allowable Lifting Load

**Figure 2:**
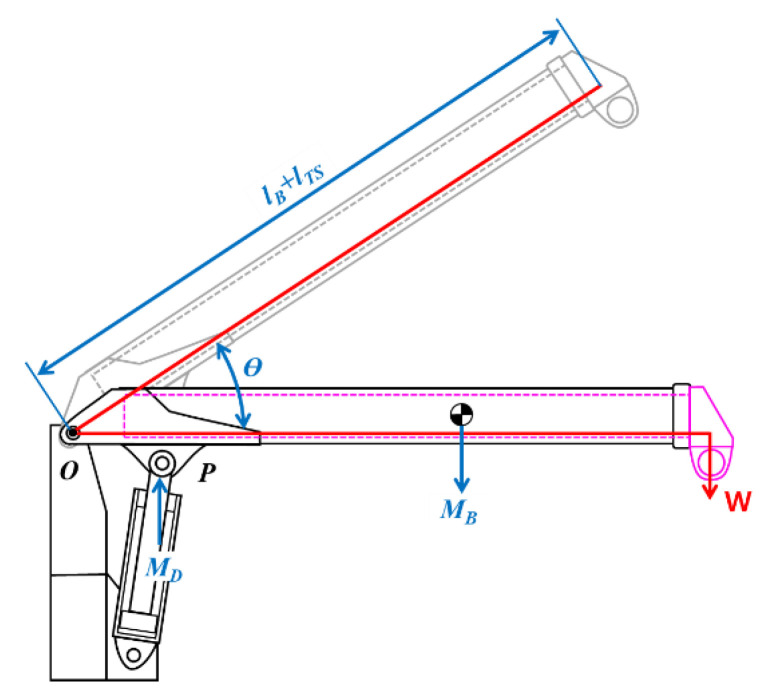
Free-body diagram for the cargo crane analysis.

The motions of a mobile crane (e.g., ups and downs of the boom, boom extension and shortening, etc.) are dictated by the extrusion and intrusion of telescopic and derrick cylinders. The relationship between the allowable lifting load that dictates the momentary angle and the length changes in the boom and the pressure occurring in the hydraulic cylinder is modeled into a free-body diagram in Figure 2 to establish the mathematical formula.

The boom rotates around the center point denoted to *O* by dictating the extrusion and intrusion of the derrick cylinder rod. The load balancing the moments at the *O* point is formulated in Equation (1):

(1) $$W=\frac{M_{D}-M_{B}}{\left(l_{B}+l_{TS}\right)\cos\theta }$$

where, *W* is the lifting load acting on the boom; $M_{D}$ is the moment generated by the derrick cylinder weight around the center point *O*; $M_{B}$ is the moment generated by the boom weight; *Ɵ* is the boom angle; $l_{B}$ is the boom default length, and $l_{TS}$ is the final length of the boom dictated by the telescopic cylinder extrusion.

**Figure 3:**
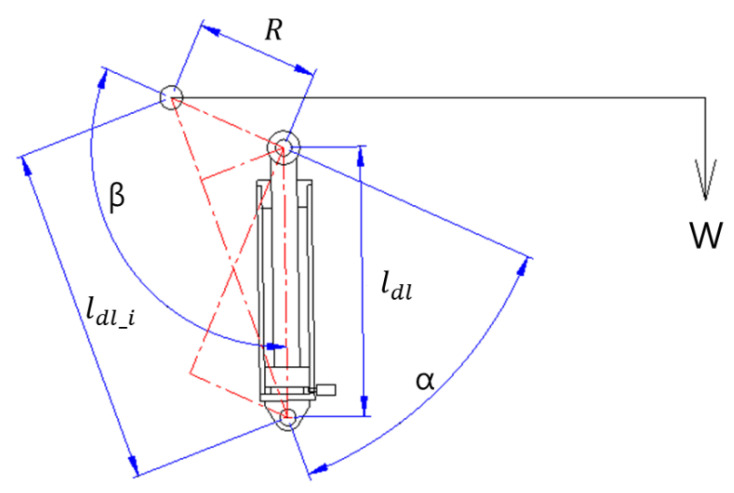
Dimensions of the derrick cylinder moment.

The locations on the spatial coordinate of each component are varied by manipulating the derrick cylinder. Figure 3 depicts the dimensional changes in the components attached to the derrick cylinder. The moment ($M_{D}$) generated by dictating these changes is formulated in Equation (2).

(2) $$M_{D}=\frac{\pi D^{2}R\Delta P\left|\cos\left(90-\beta \right)\right|}{4}$$

where, *D* is the diameter of the derrick cylinder rod; *R* is the linear distance between the rotation center point of the boom and the end pin connecting one end of the derrick cylinder rod; Δ*P* is the pressure generated on the derrick cylinder tube; and *β* is the angle between the virtual straight line (*R*) and the derrick cylinder. *β* was obtained by inputting the mechanical dimensions in Equation (3). Note that the existing method, by which every dimension was directly measured using sensors, was replaced by Equations (2) and (3).

(3) $$\beta =180-\left(a+\theta \right)-tan^{-1}\left(\frac{R\sin\left(\alpha +\theta \right)}{l_{dl\_i}-R\cos\left(\alpha +\theta \right)}\right)$$

where, $l_{dl\_i}$ is the straight distance virtually connecting the rotation center point of the boom (i.e., *O*) and the end pin connecting one end of the derrick cylinder tube; α is the angle between the virtual straight line (*R*) obtained when the derrick cylinder tube is maximally intruded and the $l_{dl\_i}$.

**Figure 4:**
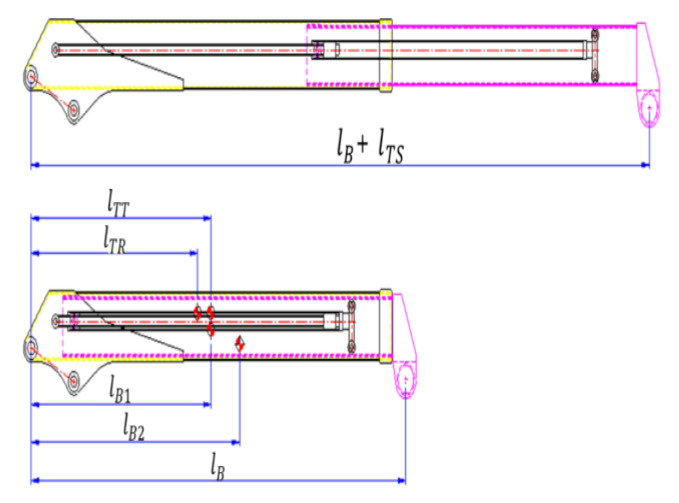
Dimension of the boom moment.

Mechanical variables, such as the boom length extended by manipulating the telescopic cylinder and the gravitational center of the boom, are defined in Figure 4. The moment ($M_{B}$) generated by the boom weight was computed by inputting the values of these variables in Equation (4).

(4) $$M_{B}=[W_{B1}l_{B1}+W_{B2}\left(l_{B2}+l_{TS}\right)+W_{TR}l_{TR}+W_{TT}\left(l_{TT}+l_{TS}\right)]\cos\theta$$

where, $W_{B1}$ and $W_{B2}$ are the weights of the first and second booms, respectively; $l_{B1}$ and $l_{B2}\;$are the gravitational center coordinates of the first and second booms, respectively; $W_{TR}$ and $W_{TT}$ are the weights of the telescopic cylinder tubes and rods, respectively; and $l_{TR}$ and $l_{TT}$ are the gravitational center coordinates of the telescopic cylinders. The allowable lifting load acting momentarily on the mobile crane is defined into a mathematical model (Equation (5)) by substituting the corresponding variables in Equation (1) with the Equations (2)–(4).

(5) $$W=\frac{\pi D^{2}R\Delta P\left|cos\left(\left(\alpha +\theta \right)+tan^{-1}\left(\frac{Rsin\left(\alpha +\theta \right)}{l_{dl\_i}-Rcos\left(\alpha +\theta \right)}\right)-90\right)\right|}{\left(l_{B}+l_{TS}\right)cos\theta }\;\;-\;\frac{W_{B1}l_{B1}+W_{B2}\left(l_{B2}+l_{TS}\right)+W_{TR}l_{TR}+W_{TT}\left(l_{TT}+l_{TS}\right)}{\left(l_{B}+l_{TS}\right)}$$

### 3.2. Computational Procedure for the Lifting Capacity Analysis by Considering the Pressure and Boom Angle

The eAML imports the mobile crane parameters provided by the equipment manufacturer into a computational workspace (Table 2).

Using the crane, the method analyzes the lifting capacity involved in changing the pressure and the boom angle using the lifting load computational model shown in Equation (5). Table 3 shows the set of allowable lifting loads obtained by manipulating the combination of the pressure on the boom and the boom angle. Table data confirm that the crane may safely operate at all angles without being involved in a rollover only if the lifting load and the pressures on the boom are less than or equal to 160 kgf and 40 kgf/cm^2^, respectively.

**Figure 5:**
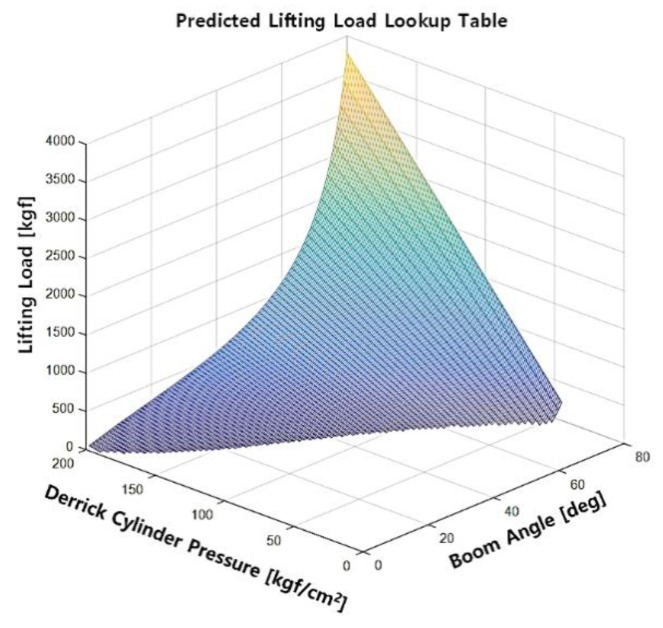
Predicted lifting load (lookup table).

The lifting load table in Table 3 was modeled into the three-dimensional (3D) graph shown in Figure 5 to increase data reusability in the digital computation. Given the derrick cylinder pressure and the boom angle, the digitized graph computes the mechanical dimension of the mobile crane (i.e., extruded length and boom angle) and returns the allowable lifting load. The eAML facilitates the acquisition of the corresponding digitized 3D lifting load graph of a mobile crane when the mobile crane type changes only if Table 2 is updated with the corresponding equipment parameters provided by the equipment manufacturer.

## 4. Algorithm Controlling the eAML

**Figure 6:**
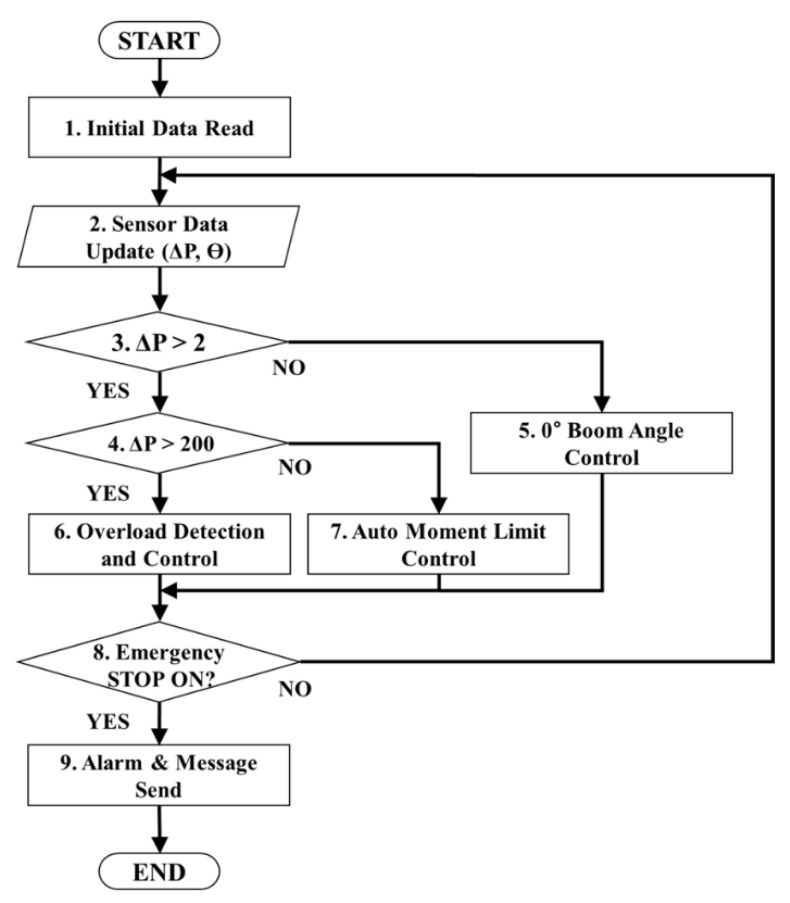
eAML control algorithm.

Figure 6 displays the algorithm controlling the crane operation using the mathematical model for the lifting load. The algorithm consists of three modules: manipulating the boom angle at 0°; detecting and controlling the overload, and controlling the eAML. The eAML reads the mechanical dimensions of the crane (i.e., *D*, *R*, α, $l_{dl\_i}$, $W_{B1}$, $W_{B2}$, $W_{TR}$, $W_{TT}$, $l_{B1}$, $l_{B2},\;l_{TT}$, $l_{TS}$, $l_{B}$, and $l_{TR}$) as the default values in Step 1 of Figure 6. In Step 2, it updates the pressure value obtained from the pressure sensor installed at the bottom (i.e., tube) of the derrick cylinder and the angle value obtained from the angle sensor installed on the boom. Subsequently, it identifies if the derrick cylinder is completely intruded in Step 3. The method confirms that the boom angle reaches 0° if the value obtained by the pressure sensor installed at the bottom tube of the derrick cylinder is less than 2 kgf/cm^2^, which indicates a completely downed boom. The pressure on the derrick cylinder cannot be detected once the derrick cylinder is completely intruded. Therefore, determining whether the boom reaches 0° or not may be confirmed by checking if the minimum pressure detected by the pressure sensor installed at the bottom tube of the derrick cylinder reaches 2 kgf/cm^2^. When the boom reaches 0°, the boom angle is adjusted to 0° in Step 5. However, if the pressure of the derrick cylinder exceeds 200 kgf/cm^2^, the overload detection and control is performed in Step 6. When the pressure of the derrick cylinder is greater than 2 kgf/cm^2^ and less than 200 kgf/cm^2^, the auto moment limit control is performed in Step 7. If an emergency stop is not performed in Step 8, Steps 2 to 8 are executed repeatedly. Otherwise, the alarm and message are sent to the crane operator in Step 9.

### 4.1. Manipulating the Boom Angle at 0°

**Figure 7:**
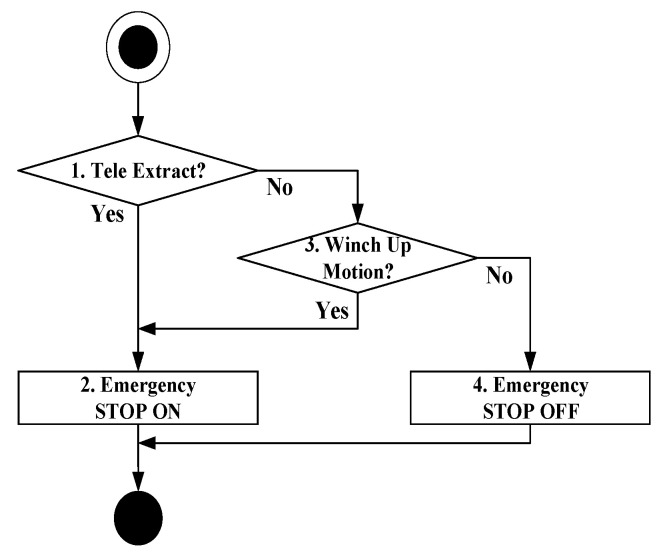
Zero degree boom angle control algorithm.

Figure 7 depicts the procedure for controlling the boom angle from 0°. The method verifies if the crane controller commands the boom extrusion in Step 1. If the boom extrusion command has already been issued out, it orders to stop the boom extrusion in Step 2; otherwise, it checks if a winch up command has already been issued out to lift the load in Step 3. The method limits the boom extrusion and the winch rise to secure the crane safety, because it cannot compute the allowable lifting load using the mathematical model shown in Equation (5), unless the derrick cylinder pressure is detected.

### 4.2. Detecting and Controlling Overload

**Figure 8:**
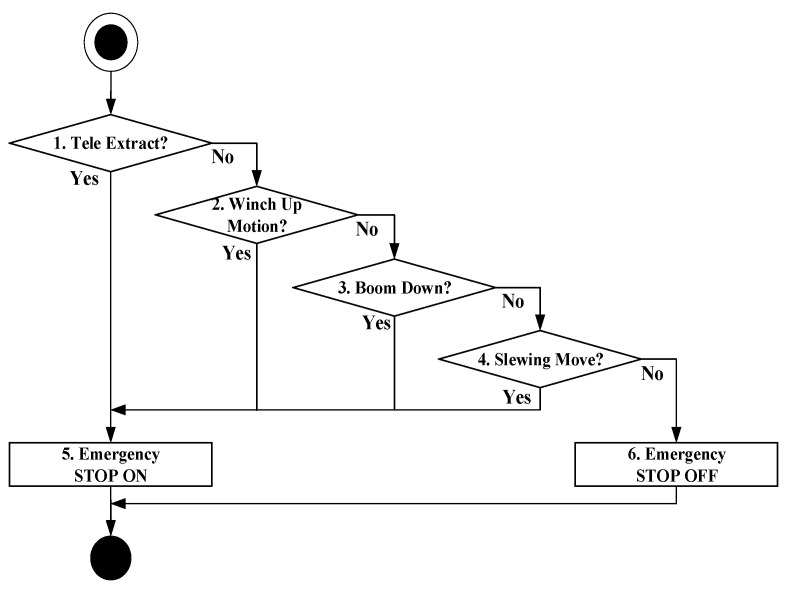
Overload detection and control algorithm.

The crane shall make an emergency stop by restricting the motions involved in the extruding boom, rising winch, downing boom, and rotating crane to secure the crane safety if the pressure measured in the derrick cylinder exceeds the maximum allowable pressure of 200 kgf/cm^2^ (hereinafter referred to as overload). For this purpose, the module that detects and controls the overload is implemented in accordance with the algorithm in Figure 8. If the method detects an event at which the pressure detected in the derrick cylinder exceeds the maximum allowable pressure, it checks if any of the commands associated with the extruding boom, rising winch, downing boom, and rotating crane are issued out in Steps 1 to 4, respectively. The crane makes an emergency stop if any of these commands is detected.

### 4.3. Controlling the eAML

**Figure 9:**
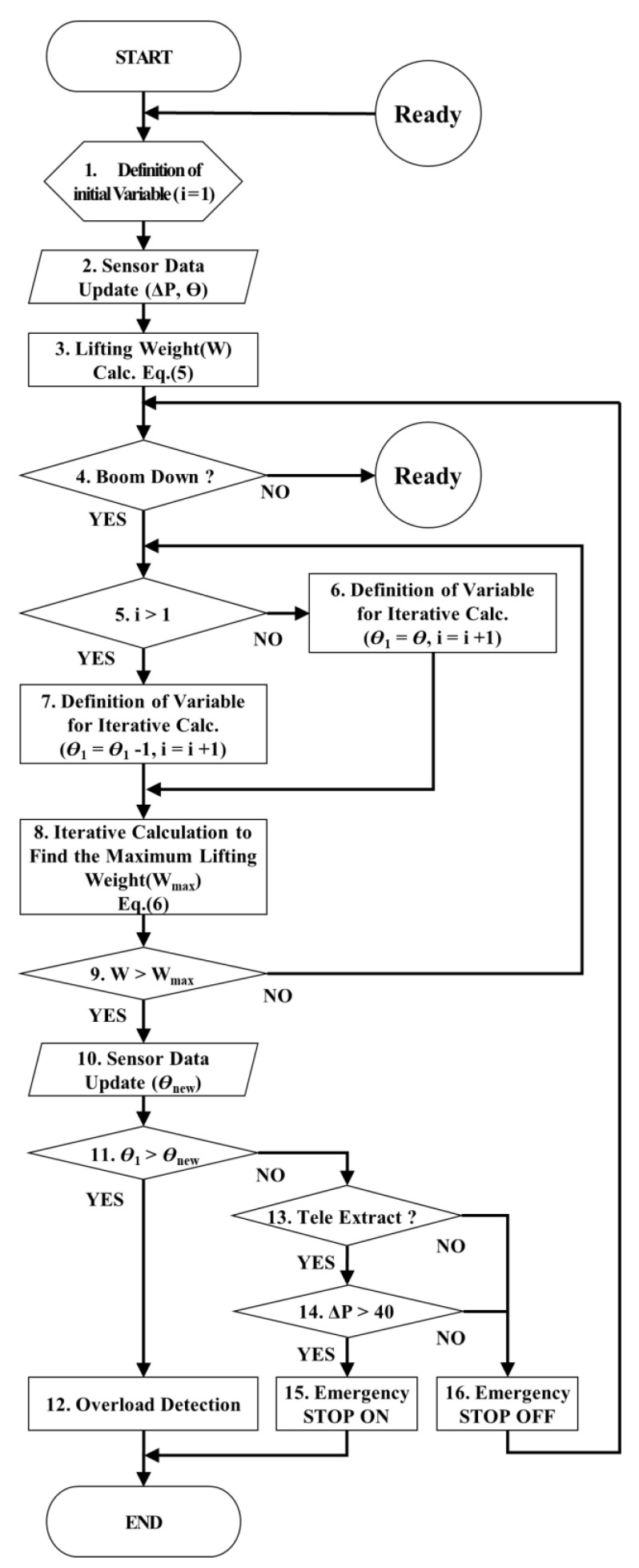
Auto moment limiter control algorithm.

The module that controls the eAML safely manipulates the boom components using the output data obtained by the two modules (i.e., one verifying if the derrick cylinder is completely intruded and the other determining if the crane has overloaded) described in the previous sections. The control algorithm of eAML is illustrated in Figure 9.

After inputting the values associated with the boom angle and the derrick cylinder pressure in Step 2, the method computes the current lifting load (*W*) using Equation (5) in Step 3 and determines if a command to down the boom is input in Step 4. It confirms if the initial variable (i) is greater than 1 in Step 5. If it is less than or equal to 1, the maximum lifting load is calculated by using the value of the boom angle obtained by the angle sensor attached to the boom in Step 8. If the initial variable is greater than 1, the maximum lifting load is calculated using the value of the boom angle reduced by 1° ($\theta_{1}$ = $\theta_{1}$ − 1) in Step 8. It then calculates the maximum allowable lifting load by assuming that the threshold value of the moment is given to the crane in Step 8.

The derrick cylinder pressure reaches the maximum if the maximum lifting load is applied to the boom. Therefore, Equation (5) is transformed to Equation (6) by substituting ΔP with 200 kgf/cm^2^. The maximum lifting load ($W_{max}$) is computed using Equation (6) in case of a downed boom. The computation using Equation (6) is repeated iteratively, while decreasing the boom angle value through a user-defined decrement (i.e., 1°, $\theta_{1}$ = $\theta_{1}$ − 1) in Step 9.

(6) $$W_{max}=\frac{200\pi D^{2}R\left|\cos\left(\left(\alpha +\theta_{1}\right)+\tan^{-1}\left(\frac{R\sin\left(\alpha +\theta_{1}\right)}{l_{dl\_i}-R\cos\left(\alpha +\theta_{1}\right)}\right)-90\right)\right|}{\left(l_{B}+l_{TS}\right)\cos\theta_{1}}\;\;-\;\frac{W_{B1}l_{B1}+W_{B2}\left(l_{B2}+l_{TS}\right)+W_{TR}l_{TR}+W_{TT}\left(l_{TT}+l_{TS}\right)}{\left(l_{B}+l_{TS}\right)}$$

The lowest allowable angle $\theta_{1}$ for the boom to descend with the load was determined by comparing the maximum lifting load $W_{max}$ with the current lifting load *W*. $\theta_{1}$ is the angle at which the boom can safely descend to its maximum. The method checks if the angle value $\theta_{new}$ of the boom measured by the sensor in real time is less than $\theta_{1}$ in Step 11. If it is, the boom extrusion and the winch rise are limited in Step 12. If the pressure on the derrick cylinder tube is less than or equal to 40 kgf/cm^2^ in Step 14, the emergency stop switch will not be turned on in Step 15 using the lifting capability table that provides the allowable working ranges (Table 3). The boom may safely be raised or downed in the entire angle range.

## 5. Verification Experiment

A set of controlled experiments are performed to address the potential risk (i.e., boom drop) attributed to replacing the expensive stroke sensors and load cells with the economical sensors, formula, and algorithm in this method validation section. This section verifies if the crane boom may suddenly drop, while the crane boom is descending from 70° to 0° in each case of with and without eAML along with sensors. It elaborates how this method eliminates the lack of warning information and mitigates the potential risk (i.e., boom drop) attributed to replacing the expensive stroke sensors and load cells with the economical sensors, formula, and algorithm.

### 5.1. eAML Simulation Using the Maximum Lifting Load Prediction Method

**Figure 10:**
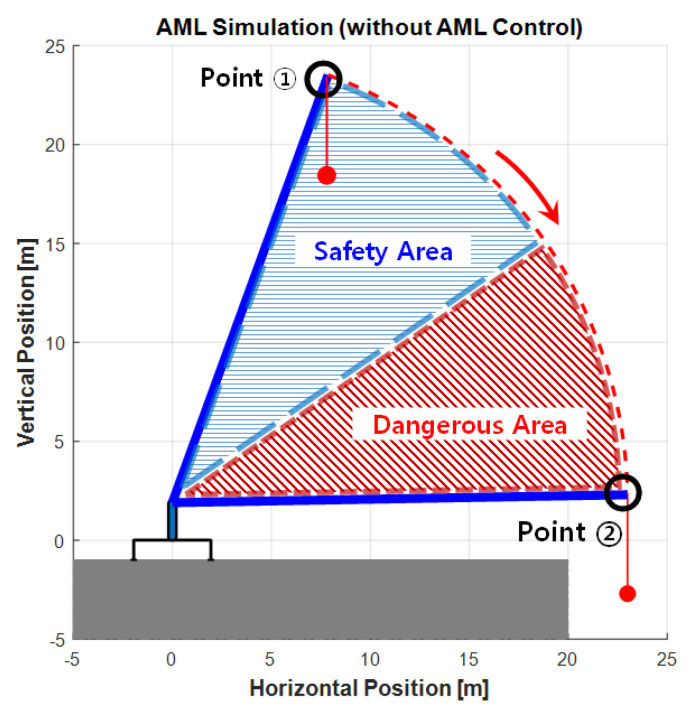
AML simulation outputs without control.

**Figure 11:**
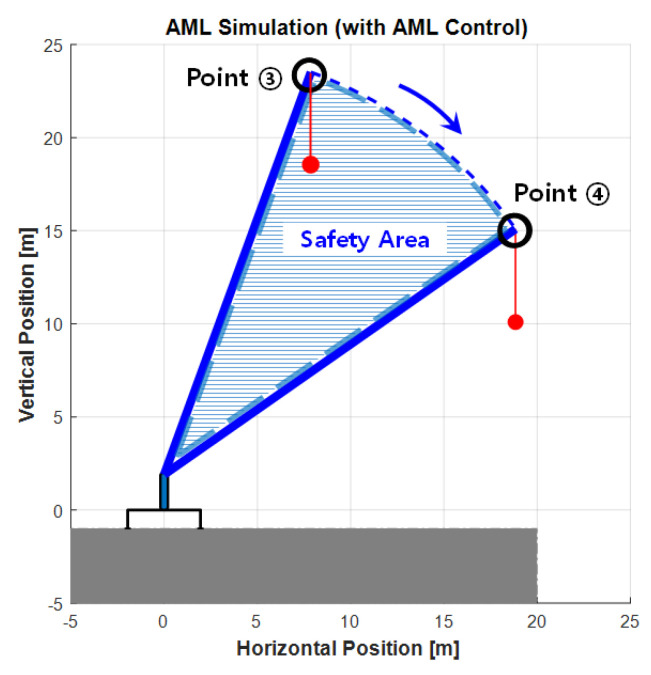
AML simulation outputs with control.

A simulation experiment that models the lifting load operation using the eAML control algorithm was performed using MATLAB version R2020a (2020). The method validity was then verified by comparing the simulation outputs with the results obtained from the real-world mobile crane operation experiment. The method confirmed if the boom in the simulation experiments was controlled, while the boom angle descended from 70° to 0°, with the crane boom being maximally extruded and the user-defined weight being hooked (e.g., 500 kgf) as the load. The operation lowering the crane boom was simulated with two options: (1) executing the simulation without applying the eAML algorithm and (2) executing the simulation applying the algorithm. Figure 10 and Figure 11 show the outputs obtained by the two options, respectively. In the first case, the boom descended without stopping from Point 1 (initial angle: 70°) to Point 2 (final angle: 0°). In the second case, the boom started from Point 3 with an initial angle and descended only to Point 4. This result provides admissible evidence that the eAML algorithm can control the boom to no longer descend when approaching the unsafe angle zone.

**Figure 12:**
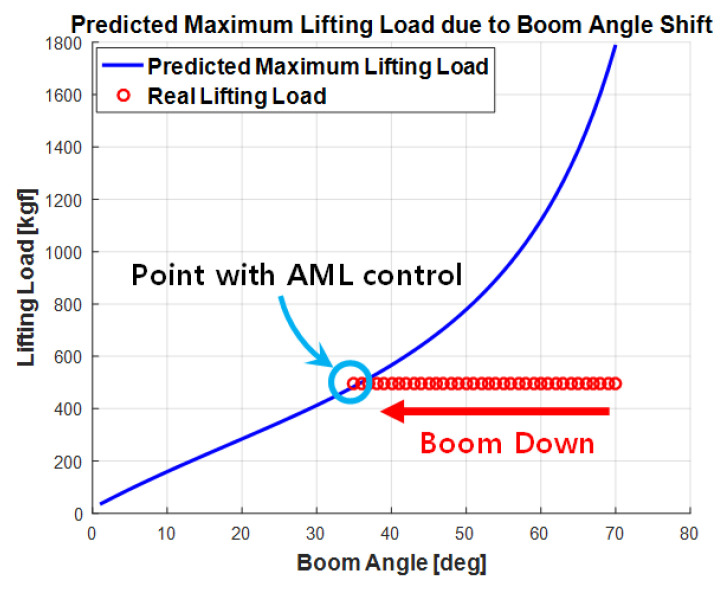
Simulation outputs given the predicted maximum weight and heavy object movement.

The best-fit curve that modeled the moving path of the 500 kgf load and the $W_{max}$ value was identified using the curve fitting tool (Figure 12) when the boom descended during the eAML algorithm application. That is, the lesser the descending boom angle, the smaller the allowable lifting load obtained by dictating the exponential function shown in Equation (7). The eAML algorithm prevented the boom from descending when the lifting load was equal to $W_{max}$.

(7) $$W_{max\_bc}=127.6e^{0.03681x}-136.9e^{-0.1992x}$$

**Figure 13:**
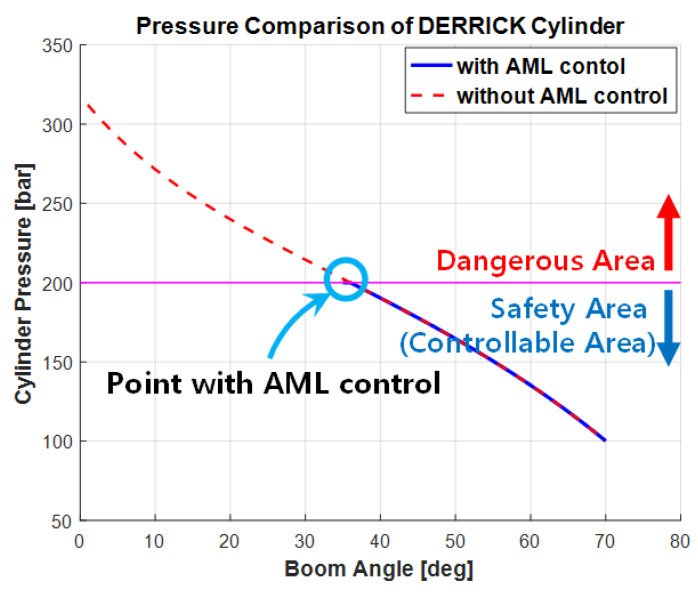
Simulation outputs of the derrick cylinder pressure.

Figure 13 exhibits the simulation outputs obtained by manipulating the pressure on the derrick cylinder tube and the boom angle for the two cases in which the eAML algorithm was applied and not, respectively. The threshold pressure on the hydraulic system in operation was 200 kgf/cm^2^. An operated pressure exceeding the maximum pressure causes the boom to descend without stopping to 0°, resulting in an accident. The 200 kgf/cm^2^ threshold classified the boom operating range into danger and safe zones that are controllable. The operating ranges denoted by the dashed lines in the figure represent the danger zone in which the boom angle continued to descend, because the eAML was not used, and the safe zone in which the boom angle stopped within the threshold pressure because the eAML was used.

**Figure 14:**
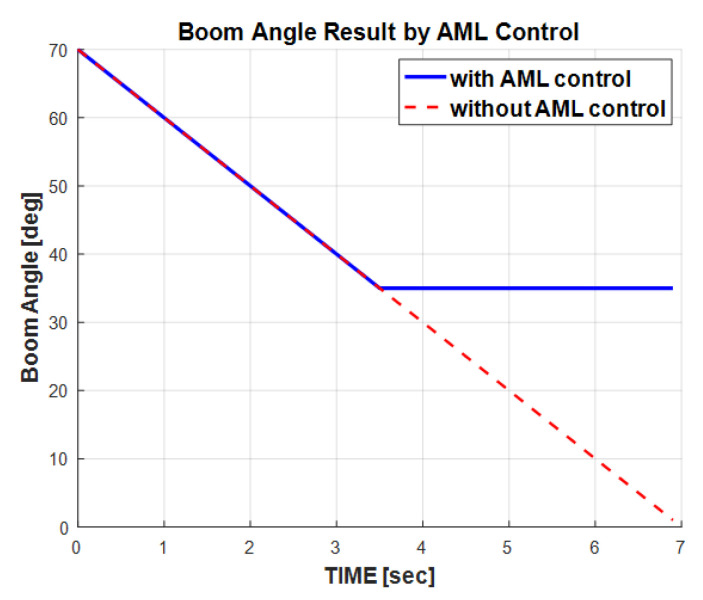
Boom angle simulation outputs with AML control.

Figure 14 presents the eAML controllability to the boom angle. The boom angle descended from the initial 70° to the final 0° when the eAML was not used. In contrast, the boom angle descended from the initial 70° only to the final 35° when the AML was used. In other words, the eAML controlled the boom within the range in which the lifting load did not exceed the $W_{max}$ value. This simulation outputs provide admissible evidence that eAML prevents the crane from overloading.

### 5.2. Validation of the eAML

#### 5.2.1. Experiment Method

**Figure 15:**
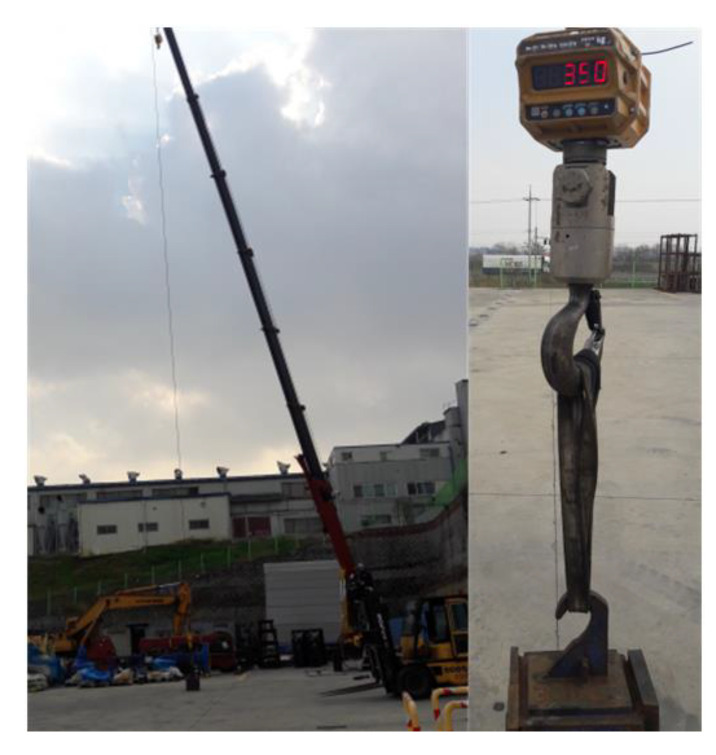
Experiment and lifting load scale.

A real-world physical experiment was performed using a midsize mobile crane (see Figure 15) having the machine parameters shown in Table 2, which includes the maximum pressure of the hydraulic pump, which is 210 kgf/cm^2^, to validate the method. The experiment consists of two folds, i.e., the performance reliability test and the robustness against to the external variable (i.e., wind). The subject and environment of the experiment are shown in Table 4. One was tested by lifting 30 times repetitively each weight out of various weights (i.e., 390, 500, 1000 kgf); the other by repetitively creating wind 30 times obtained by selecting the date on which different average wind speeds occurs for each weight. The crane equipped with the eAML was operated after defining the environmental attributes shown in Table 4. For example, after locating the lifting load of 390 kgf and the lifting aids (i.e., hooks, auxiliary hooks, and scales) of 110 kgf to a spatial coordinate in which the boom angle was 70°, the boom is decreased to 0° to see if it stopped in an intermediate degree between these two.

#### 5.2.2. Experiment Outputs

##### Validation of Performance Reliability

**Figure 16:**
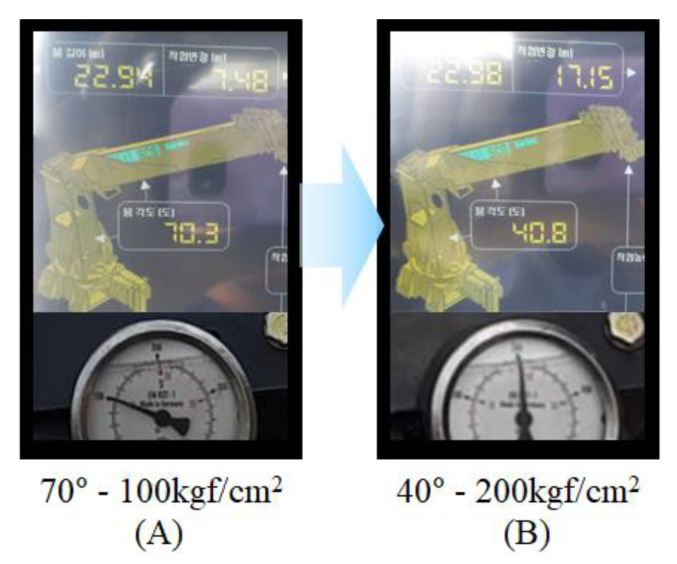
Experiment outputs: boom angle and derrick cylinder pressure.

The performance of eAML is verified by evaluating the reliability of the boom angle control observed after conducting experiments repeatedly to lift each of the various weights. Given the lifting load 390 kgf, the experiment outputs are shown in Figure 16A,B. The former represents the pressure value (i.e., approximately 100 kgf/cm^2^) acting on the derrick cylinder tube when the initial boom angle is 70°; the later indicates the boom angle (i.e., 40°) and the pressure value (i.e., approximately 200 kgf/cm^2^) acting on the derrick cylinder tube at the moment the boom is controlled by the AML. The boom stops when the threshold of pressure on the derrick cylinder reaches 200 kgf/cm^2^. The gauge at the bottom of Figure 16 depicts the boom angle and the pressure on the derrick cylinder tube obtained when the eAML was used. Figure 16A shows the pressure on the derrick cylinder tube and the boom angle obtained when the initial boom angle was 70°. Figure 16B displays the pressure and the angle obtained when the boom was controlled by the eAML. The pressure was approximately 100 kgf/cm^2^ when the boom angle was 70°. This value was equivalent to the allowable lifting load provided in the lifting load in Table 2. The pressure and the angle obtained when the boom stopped due to the eAML control were approximately 200 kgf/cm^2^ and 40°, respectively, confirming that the pressure value may be equivalent to the lifting load (Table 2).

**Figure 17:**
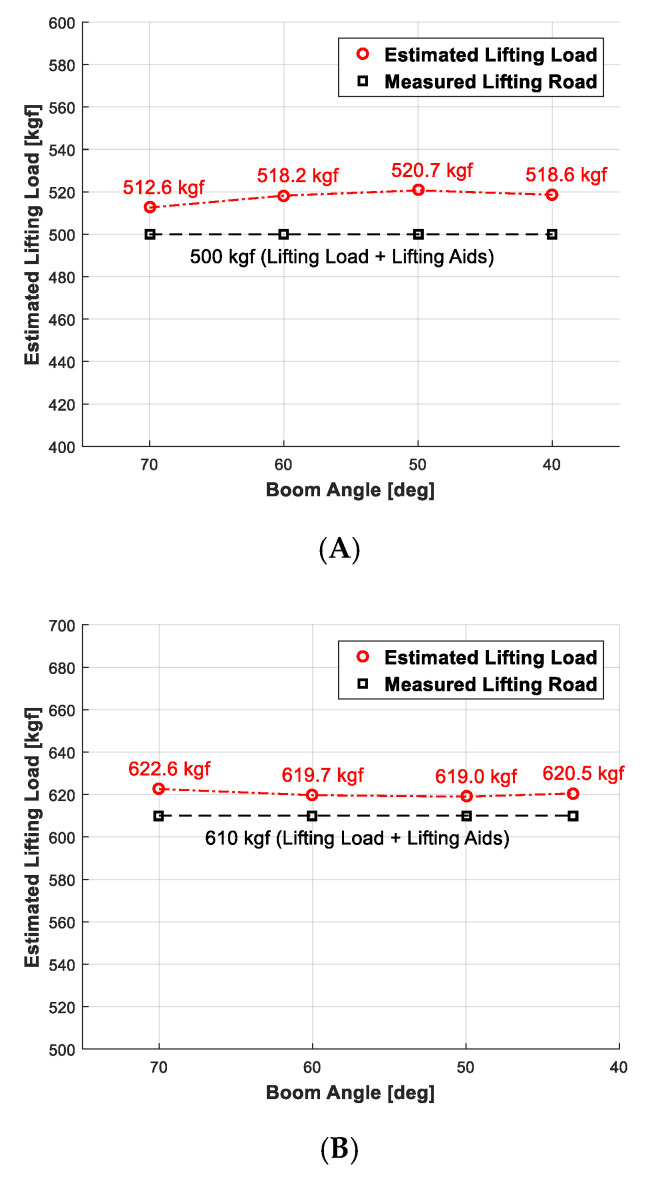

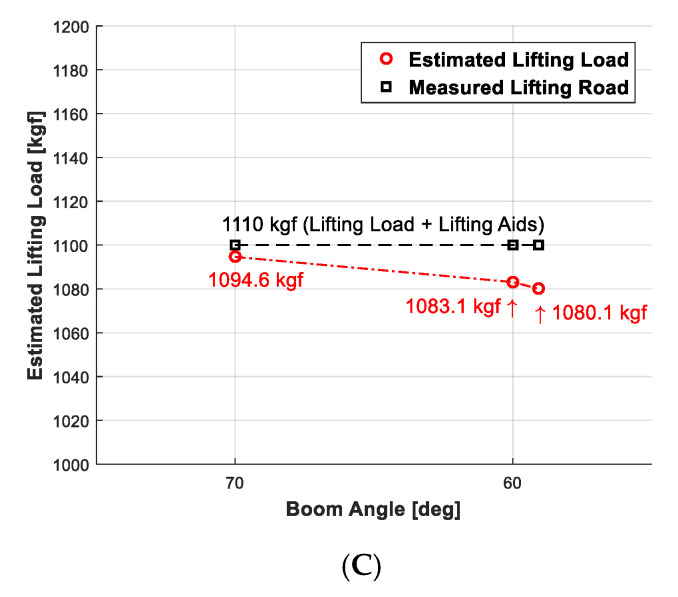
Experiment outputs (estimated lifting load and measured lifting load). (**A**) 390 (+110) kgf; (**B**) 500 (+110) kgf; (**C**) 1000 (+110) kgf.

The performance reliability evaluation of lifting loads obtained by conducting 30 iterations of experiments compared to the estimated lifting loads shown in Table 3 is presented in Figure 17. The outputs in Figure 1, which were obtained while lowering the angle of the boom by 10° from 70° to 0° given the lifting load is 390, 500, and 1000 kgf, respectively, compare the lifting load estimated using the load scale with that available in Table 3. Any lifting load that dictates the pressure value of the derrick cylinder may be found using the straight interpolation method when it is not available in Table 3.

**Figure 18:**
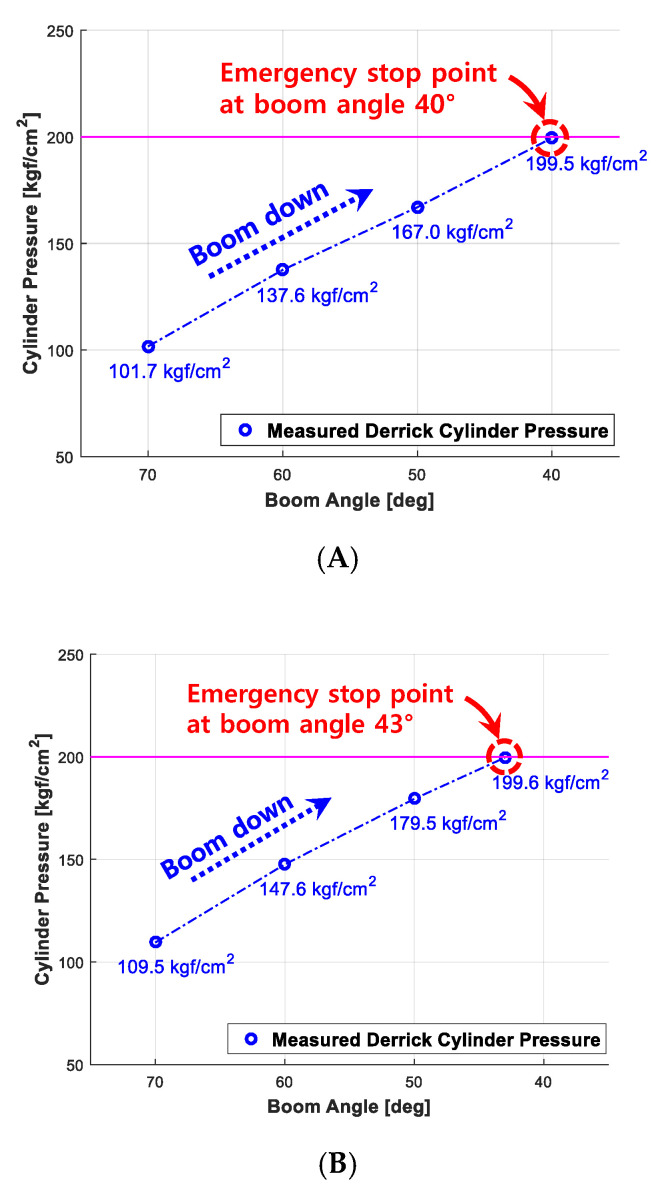

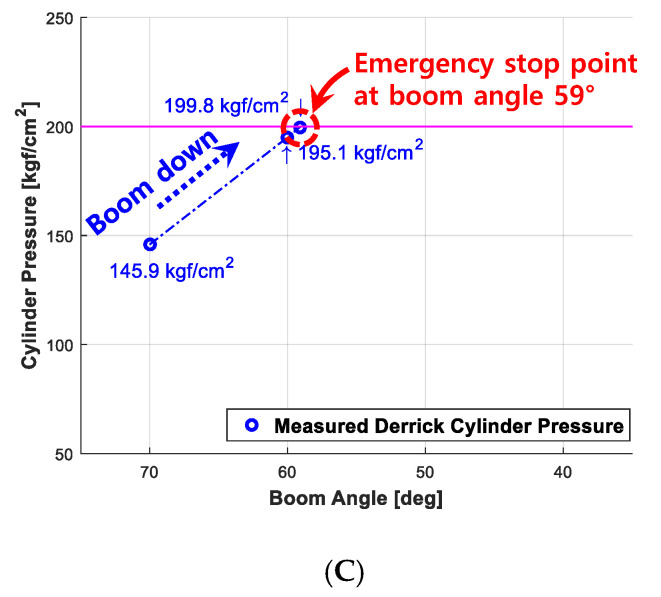
Experiment outputs (controlled boom angle and derrick cylinder pressure). (**A**) 390 (+110) kgf; (**B**) 500 (+110) kgf; (**C**) 1000 (+110) kgf.

The performance reliability outputs, which are obtained when the boom angle is controlled using eAML algorithm (i.e., the threshold of pressure on derrick cylinder; 200 kgf/cm^2^, the boom descent stops upon arrival), are shown in Figure 18. Given the lifting loads 390, 500, and 1000 kgf, respectively, it provides the boom angle measured and the pressure on the derrick cylinder which are obtained, while lowering the boom angle by a 10° from 70° to 0°. It denotes the angle of the boom and the threshold of pressure on the derrick cylinder obtained at the moment in which the boom is controlled and stopped by eAML. The figures in each graph are the arithmetic average of the outputs obtained by performing repetitively 30 times experiments on each lifting load.

##### Validation of Robustness to Wind

**Figure 19:**
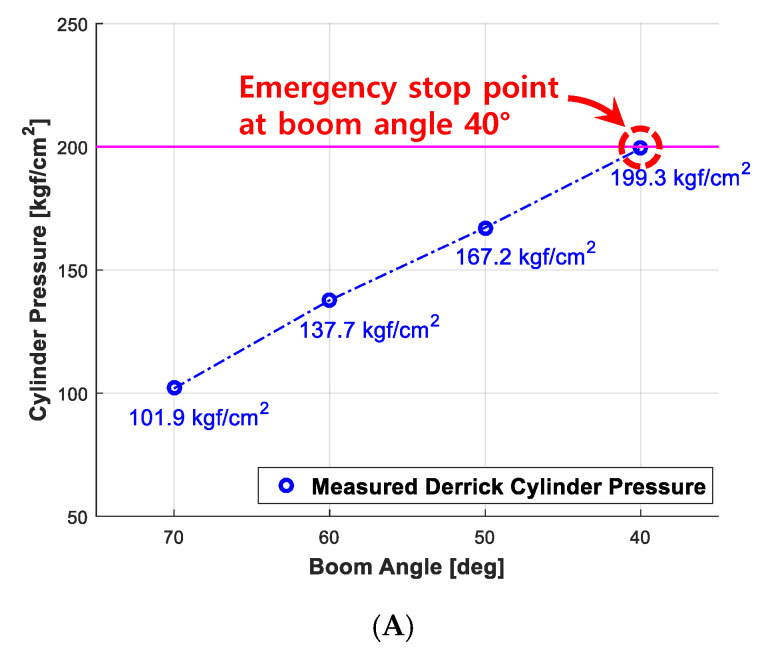

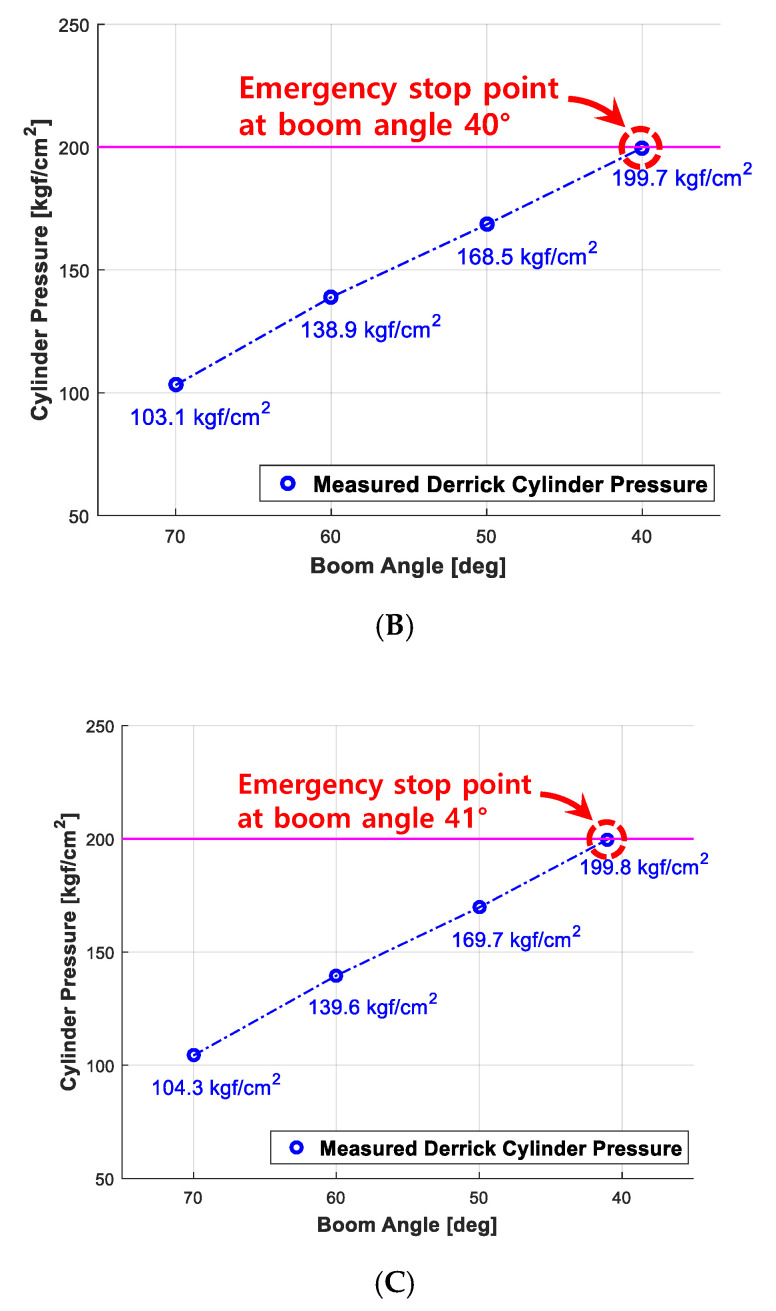
Experiment outputs obtained when controlling boom angle and derrick cylinder pressure in each wind speed. (**A**) 1~2 m/s; (**B**) 3~4 m/s; (**C**) 5~6 m/s.

Lifting operation using a cargo crane is affected by external environment factors such as wind. At the very moment when the load is swung by the wind, the static load is converted to the dynamic load, which acts on the boom, hence, resulting in a greater moment. It means that the derrick cylinder demands greater pressure. Certainly, it justifies developing an eAML algorithm that effectively controls the boom angle attributed to wind effects. The boom angle and the pressure on the derrick cylinder, which are obtained by a rigorous real world experiment when the boom handles the load of 390 kgf, provides admissible evidence regarding how effectively eAML controls the boom angle in responding to the wind effects. The validation experiments are performed by changing the wind speed from the minimum of 1 m/s to the maximum of 6 m/s and downing the angle of the boom by 10° per each down from 70° to 0°. The threshold value of boom angle and that of pressure on derrick cylinder, which are obtained at the very moment the boom is controlled and stopped by eAML, are explicitly denoted in Figure 19. The figures in each graph represent the arithmetic average of the outputs obtained by 30 experiments performed repetitively on each lifting load. Experiments on wind effects confirms that the wind speed of 5 to 6 m/s affects to the pressure on the derrick cylinder and causes the boom to stop before 1° (i.e., 41°).

### 5.3. Predicting the Allowable Lifting Loads Using eAML Simulation Outputs

**Figure 20:**
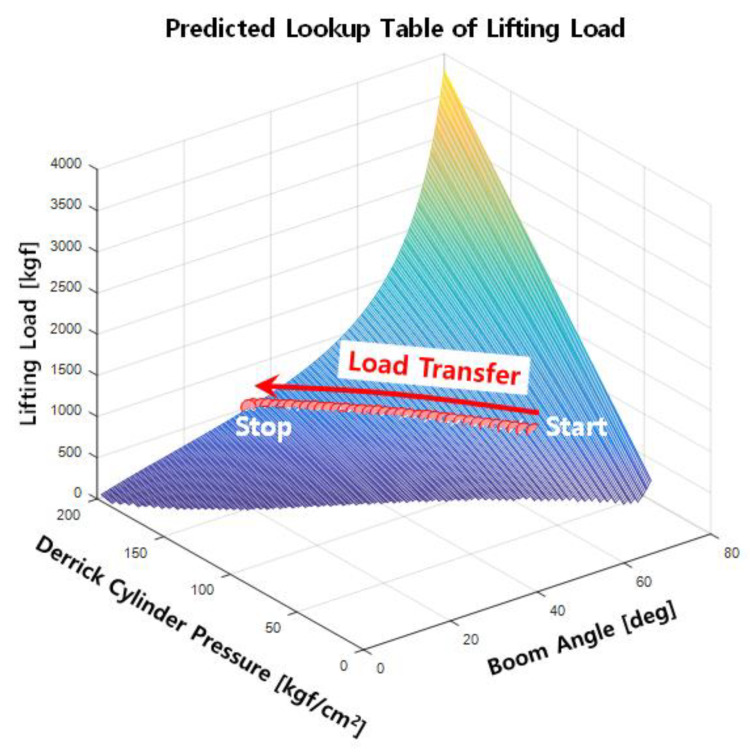
Experiment outputs: load transfer.

Figure 20 illustrates the allowable lifting loads that can be safely handled when the wind speed is less than 6m/s. The boom with the eAML control stopped at 40° when the pressure was close to 200 kgf/cm^2^. The experiment with the eAML provided empirical evidence that it can safely handle lifting loads. As mentioned in the previous section, the boom stops 1° before the threshold of 40° at a wind speed of 6 m/s. The graphs modeling the allowable lifting loads may be expended by identifying the thresholds, while increasing the wind speeds. This result provides admissible evidence that the eAML algorithm can control the boom to no longer descend when approaching the unsafe angle zone.

## 6. Conclusions

This study presented a new AML control algorithm, called the eAML, which reduces the unit cost, while retaining the performance of existing AMLs that identifies and controls in real time any overload to the mobile crane. The main contribution of the study is the mathematical formula and the control algorithm implemented in eAML. The eAML identifies the allowable lifting load and the maximum load that can be safely handled using only the pressure on the derrick cylinder tube and the boom angle. In addition, the algorithm controls the boom by keeping the pressure generated by the hydraulic system operation lower than the threshold pressure when the boom descends, which prevents the boom from overloading. The mathematical methods that formulates the allowable and the maximum lifting loads of the mobile crane and the eAML algorithm that controls the boom by implementing the method are beneficial to the crane safety community. The eAML implemented using MATLAB provides a tool to perform simulation verification to ensure that the boom controls the overload depending on the application of the control algorithm. The experiments involved in the performance reliability and the robustness to wind confirm the reliability of the method. Repetitive experiments conducted at various wind speeds confirm that eAML is robust to environmental effects. The method proven by a comparative empirical study with a simulation experiment herein and a real-world crane operation is currently being applied to the SCS887S crane of Korea Fisheries Heavy Industries. The method contributes to eliminating the socioeconomic limitations associated with the use of AMLs by remarkably reducing the production cost. The eAML may encourage small self-employed owners to access it, ensuring safety in response to momentary and transitory changes.

## Figures and Tables

**Table 1:** Comparison of the existing AML and economical auto moment limiter (eAML) systems.

Type		Advantages	Disadvantages
Existing AML	Pressure-type AML	Lower price than the load cell-type AML	Undetectability of the overload pressure at 0° Poor durability
	Load cell-type AML	Fall detection of derrick Detectability of pressure overload at 0°	Load cell sheave deformation High price Possibility of external overlap Poor durability
eAML	Low-cost AML	Fall detection of derrick Detectability of pressure overload at 0° Low price	Necessity of initial calibration

**Table 2:** Midsize cargo crane (7.5 ton) parameters.

Parameter	Notation	Value	Unit
Internal diameter (derrick cylinder)	*D*	17	cm
Rotational radius (derrick cylinder)	*R*	588.8	mm
Tube pressure (derrick cylinder)	Δ*P*	0–200	kgf/cm^2^
Assembly angle (derrick cylinder)	*α*	34	deg
Luffing angle (boom)	*Ɵ*	0–80	deg
Initial distance (derrick cylinder)	*l_dl_i_*	1568	mm
Initial distance (boom)	*l_B_*	19,995	mm
Tele cylinder stroke	*l_TS_*	2970	mm
Weight of the first boom	*W_B_*_1_	460	kgf
Distance to center of mass (first boom)	*l_B_*_1_	2650	mm
Weight of the second boom	*W_B_*_2_	1200	kgf
Distance to center of mass (second boom)	*l_B_*_2_	10,879	mm
Rod weight (telescopic cylinder)	*W_TR_*	80	kgf
Distance to center of mass (telescopic cylinder rod)	*l_TR_*	2246	mm
Tube weight (telescopic cylinder)	*W_TT_*	220	kgf
Distance to center of mass (telescopic cylinder tube)	*l_TT_*	7200	mm

**Table 3:** Lifting load table for the AML control of a midsize cargo crane (7.5 ton).

Lifting Load W (kgf)		Boom Angle (°) *θ*							
		10	20	30	40	50	60	70	80
Derrick cylinder pressure (kgf/cm^2^) Δ*P*	40								122
	45								237
	50								351
	55								466
	60								580
	65							46	695
	70							111	810
	75							175	925
	80							240	1039
	85						19	304	1154
	90						67	369	1268
	95						114	433	1383
	100						162	498	1497
	105					31	210	562	1612
	110					70	258	627	1726
	115					110	306	620	1841
	120				22	149	354	756	1956
	125				56	188	402	821	2070
	130				90	228	450	886	2185
	135			20	124	267	498	950	2299
	140			50	158	306	545	1015	2414
	145			81	192	346	593	1076	2528
	150		15	111	226	385	641	1144	2642
	155		42	141	260	424	688	1208	2757
	160		69	171	294	464	736	1273	2872
	165		96	201	328	503	784	1338	2987
	170	16	123	232	362	542	832	1402	3101
	175	40	150	262	396	581	880	1467	3215
	180	64	177	292	430	621	928	1532	3330
	185	88	204	322	464	660	975	1596	3445
	190	111	231	352	498	700	1023	1661	3559
	195	135	257	382	532	739	1072	1725	3674
	200	160	284	412	566	779	1119	1790	3788

**Table 4:** Experiment method and environmental attributes.

Experiment Items	Performance Reliability	Robustness to Wind
Method	Checking when the boom stops (when the maximum pressure of the derrick cylinder is 200 kgf/cm^2^) while lowering the angle of the boom from 70° to 0° (measured 30 times for each) with various weights.	Checking when the boom stops (when the maximum pressure of the derrick cylinder is 200 kgf/cm^2^) while lowering the angle of the boom from 70° to 0° (measured 30 times each) given an identical load (390 kgf) and different wind speeds.
Measurement attributes	ΔP, θ, W	ΔP, θ, W
Output variables	θ1, θc, Wmax	θ1, θc, Wmax
Experiment object and environment	Loads of 390, 500, 1000 kgf; the lifting aids (i.e., hooks, auxiliary hooks, and sensors for validation) of 110 kgf.	The threshold of wind speed with a maximum 6 m/s or less.

## Notation

*D*	internal diameter of a derrick cylinder
*l_B_*	Initial length before boom extension
*l_B_*_1_	distance from point *O* to the center of mass of the first boom
*l_B_*_2_	distance from point *O* to the center of mass of the second boom
*l_dl_*	distance after the derrick cylinder extends
*l_dl_i_*	initial distance before the derrick cylinder extends
*l_TR_*	distance from point *O* to the center of mass of a telescopic cylinder (rod)
*l_TS_*	length after boom extension
*l_TT_*	distance from point *O* to the center of mass of a telescopic cylinder (tube)
*M_B_*	moment of force caused by the boom’s own weight
*M_D_*	moment of force caused by the derrick cylinder’s own weight
*O*	rotation center of the boom
*P*	center point connecting a derrick cylinder with a boom
*R*	rotational radius of a derrick cylinder
*W*	lifting load of a cargo crane
*W_B_*_1_	weight of the first boom
*W_B_*_2_	weight of the second boom
*W_max_*	maximum lifting load of a crane
*W_max_bc_*	best-fit curve of the maximum lifting load
*W_TR_*	rod weight of a telescopic cylinder
*W_TT_*	tube weight of a telescopic cylinder
*α*	assembly angle of a derrick cylinder
*β*	angle between the centerline of the derrick cylinder and an imaginary line connecting points *o* and *p*
Δ*P*	tube pressure of a derrick cylinder
*θ*	luffing angle of the boom
*θ*_1_	luffing angle variable to find the maximum lifting weight
*θ_c_*	luffing angle at which the boom can descend the lowest based on its current angle
*θ_new_*	luffing angle of the boom measured in real time
